# Burned aggression: the relationship between burnout and aggressive behaviour among young adults in Czechia

**DOI:** 10.3389/fpsyt.2026.1872129

**Published:** 2026-07-01

**Authors:** Ivan Sebalo, Martina Sebalo Vňuková, Martin Anders

**Affiliations:** Department of Psychiatry, First Faculty of Medicine, Charles University in Prague and General University Hospital in Prague, Prague, Czechia

**Keywords:** aggression, burnout, coping strategies, depression, emotion regulation strategies

## Abstract

**Introduction:**

Despite stress being a critical component of burnout, few studies have investigated the relationship between burnout and aggressive behaviour. Therefore, the current study aims to verify whether states of exhaustion are associated with aggression.

**Methods:**

Structural equation models were constructed using data from a representative sample of 1027 young adults (M_age_ = 24.53), almost half of whom were men (n = 507). The models revealed the relationships between burnout, aggressive behaviour, emotion regulation strategies, risky alcohol consumption, stress, and adverse childhood experiences.

**Results:**

Although there were minor differences in the pathways between men and women, both models showed a good fit for the data. Furthermore, among both men and women, the positive relationship between burnout and aggressive behaviour was mediated by maladaptive coping strategies and risky alcohol consumption. Interestingly, reliance on maladaptive emotion regulation strategies was also associated with increased depressive symptoms among women but not among men.

**Conclusion:**

The findings of this study reveal that aggressive behaviour is another negative consequence of burnout among young adults and highlight the importance of skills applied in responses to chronic stress. The implications of these findings and further results are discussed in relation to the existing literature.

## Introduction

Reactive aggression refers to impulsive, emotionally driven responses to perceived threats or frustration, while proactive aggression is deliberate, goal-oriented, and often used instrumentally (1, 2). Although there is an ongoing debate regarding the utility of this dichotomy, the indirect role of negative states is evident (3, 4). Displaced aggression, a subset of reactive aggression, involves directing aggressive responses toward an innocent target when the actual source of provocation is inaccessible (5, 6). Given that various stressors are known antecedents of reactive aggression (7–9), it is essential to examine whether burnout, a state resulting from prolonged stress, influences aggressive behaviour.

Burnout, defined as a state of emotional, physical, and cognitive exhaustion due to prolonged stress (10–12), is increasingly recognised beyond workplace settings, including among students (13). Indeed, a cross-national systematic review has highlighted that approximately 40% of undergraduate students from different disciplines report being burnt out, with future medics and engineers exhibiting the highest rates (14). Additionally, a recent meta-analysis of 44 studies during the COVID-19 pandemic found global prevalence rates of 56.3% for high emotional exhaustion and 55.3% for high cynicism among university students (15). These rates represent a marked increase from pre-pandemic estimates of 7.4-71.0%, underscoring the significant mental health burden facing this population. Given its strong associations with emotional dysregulation, anxiety, and depression (16b), burnout may predispose individuals to expressing reactive forms of aggression, though this relationship should be more extensively examined in young adult populations.

However, while stress response produces activation, which can fuel aggressive behaviour in response to a threat (17), the effect of physical exhaustion should be reversed. Most research on the link between burnout and aggression has focused on how encountering aggression increases burnout (18, 19). Nevertheless, emotional exhaustion can lead to displaced aggression. Emotional depletion due to interpersonal conflicts has been shown to be associated with aggressive conduct towards those who are unrelated to the conflict, i.e displaced aggression (20). Although the authors posit depletion of self-regulation as the explanatory variable, it was not specifically assessed in their study. Similarly, Loh & Saleh (21) have reported emotional exhaustion acting as an amplifying moderator for the effect of rude and uncivil behaviour in the workplace on retaliatory conduct of the same nature. Although this behaviour is not necessarily equated with aggression, it shares the retaliatory tendency of the proactive one. In this case, the hypothesised explanation revolves around the depletion of cognitive skills, which follows the emotional one and results in mismanaging the situation. The suspected culprit is the inability to utilise appropriate coping and emotion regulation strategies, which have been linked to aggressive behaviour (22). Thus, depletion of emotional resources from burnout might be associated with aggressive behaviour due to the strain it exerts on coping skills.

Despite the association with cognitive fatigue, emotional exhaustion does not have a strong association with response inhibition (23). However, burnout is not restricted only to emotional exhaustion; it also includes cognitive one. The latter is associated with diminished cognitive performance and, importantly, with inhibition (24, 25). Both of these functions are inherently linked to reactive aggression (17) as well as being associated with intimate partner violence (26). Cognitive dysregulation facilitates retaliatory aggression or mismanages responses to provocations and the lack of cognitive flexibility is posited among the primary mechanisms. The perpetrators continuously engage in ineffective or maladaptive conduct when faced with challenges. Although emotional and cognitive components of burnout appear to be associated with aggressive behaviour, through behavioural dysregulation, physical exhaustion can nullify this link.

Given that burnout incorporates all three, its relationship to aggression is unclear. Unfortunately, only a few studies attempted to address this issue. Tzeletopoulou et al. (27) examined mental health professionals and reported that, unlike depression, burnout was not associated with aggressive behaviour. Moreover, when investigating this link among male police officers, Queirós et al. (28) found that models including burnout – specifically depersonalisation and emotional exhaustion – can explain minor variations in physical and verbal aggression. Due to the sampling limitations of these studies, their contrasting results indicated a need for further research with more representative samples.

Tomaszek and Muchacka-Cymerman (29) have attempted to rectify this by looking at university students and reporting an association between academic burnout and aggressive behaviour, mediated by problematic phone use. Specifically, excessive use of social media and preferring digital contact to a real-life one (phone phubbing) positively mediated the effect of academic burnout on aggressiveness during COVID-19 pandemic. Given that these mediators represent coping strategies, further studies should include a wider range. Furthermore, it is important to point out that the Buss-Perry aggression questionnaire (30) used in this study assess anger and hostility in addition to verbal and physical aggression, thereby representing aggressiveness as a trait rather than aggressive conduct. Meanwhile, inspection of the link between burnout and aggression through commonly used frameworks for understanding aggressive behaviour also suggests its presence.

Both the general aggression model (GAM) (3) and the I^3^ meta-model (31) of aggressive behaviour posit that manifestations of aggressive behavioural scripts or proclivity to aggress are heavily influenced by a process where such behaviour is “approved”. Taking into account the diminishing cognitive flexibility and executive functioning resulting from the cognitive exhaustion component of burnout (25), the latter can function as a “route” in the GAM terminology or inhibition in that of I^3^. Furthermore, emotional (dys)regulation also affects decision-making process preceding aggressive conduct. The GAM places it under affect route (3). Consequently, the emotional exhaustion component of burnout, which by definition leads to dysregulation of emotions (32), can through it facilitate aggressive conduct. Furthermore, not only do these two components have a potential relationship with aggression, but they have also been shown to interact (33). Specifically, they act as amplifying moderators for each other thereby placing those lacking in both areas at the highest likelihood of aggressive conduct. Thus, due to these two components, burnout might increase the likelihood of aggression. However, physical exhaustion argues otherwise, as aggression requires energy. One way to overcome this juxtaposition and to account for burnout as a facilitator of aggression (rather than an instigator) is to include coping strategies. Their over- or underutilisation in response to burnout would be expected to influence aggression.

Indeed, coping strategies, specifically maladaptive strategies, can mediate the influence of stress on aggressive behaviour (22). Given that stress is a critical component of burnout (24), the stress management strategies can exert a direct influence on aggression. Carlo et al. (34) have shown that emotion-focused coping is associated with increased aggression, whereas problem-focused coping is associated with decreased. Similarly, Whitman and Gottdiener (35) reported an association between maladaptive coping styles and behavioural aggression. Thus, if burnout is associated with aggression, the relationship could be mediated by coping. However, one strategy should be addressed separately: alcohol consumption.

Duke’s et al. (36) meta-analysis of 32 meta-analyses reported a stable medium-sized effect of alcohol on aggressive behaviour. Since alcohol is a relatively weak drug, it is important to highlight that this relationship is most prominent at high levels of intoxication (37). However, although alcohol impairs responses to threats and stress as well as cognitive functioning, alcohol consumption will not result in aggressive behaviour among individuals without a proclivity for aggression (in I^3^ terminology) or aggression-supportive knowledge structures (in GAM terminology) (37). Thus, alcohol consumption can function as an amplifier but not the root cause.

Given that drinking alcohol is a maladaptive coping strategy for stress (38), a similar relationship is expected to be observed between alcohol consumption and burnout. Burnout has been linked to increased alcohol consumption among both students and healthcare professionals (39, 40). Consequently, since overconsumption of alcohol facilitates aggression, those who use it as a coping mechanism against burnout are expected to be aggressive.

Furthermore, since stress among university students has been shown to be associated with adverse childhood experiences (ACEs) (41), burnout is expected to have a similar relationship. Indeed, recent studies have reported that levels of burnout among physicians and nursing students are associated with ACEs (42, 43). However, this relationship has not been confirmed in young adults.

## Current study

Despite the link between two components of burnout, namely emotional and cognitive exhaustion, and models explaining aggressive behaviour there is a lack of studies investigating the potential association between these two variables. The current study aims to address this gap in understanding the negative consequences of burnout. Additionally, it furthers understanding of aggression, as the understanding of whether a construct incorporating physical exhaustion can facilitate active behaviour is intriguing. Thus, the relationship between burnout and aggression is assessed in a representative sample of young adults from Czechia. Our sampling approach included young adults across diverse contexts: full-time students, employed individuals, and those combining work and study to reflect the different lives of this age group in Czech society. This design allowed us to examine burnout’s relationship with aggressive behaviour without presupposing different mechanisms for academical and occupational burnout. We hypothesised that burnout induced by mental and psychological exhaustion will be positively associated with aggression (H1). This relationship is expected to be positively mediated by maladaptive emotion regulation strategies and risky alcohol consumption but negatively mediated by adaptive regulation strategies (H2). Moreover, burnout is expected to be positively associated with depression and anxiety symptoms (H3). Finally, we also expected the relationship between ACEs and burnout to be present in the sample of young adults (H4).

## Methods

### Sample

A representative sample of 1027 young Czech adults (aged 18 to 30 years; *M*_age_ = 24.53) was recruited. The data collection run between 1^st^ March 2023 and 30^th^ July 2023. The sample was almost equally split between males and females (n=507 and n = 520, respectively). This study was conducted in collaboration with the agency STEM/MARK, which uses Computer-Assisted Web Interviewing (CAWI). Participants were included if they were 18 to 30 years old, resided in the Czech Republic, and were fluent in Czech. Individuals were excluded if they had a severe psychiatric condition associated with substantial cognitive impairment or any legal restriction affecting their capacity to provide informed consent independently. Online informed consent was obtained prior to online data collection, and the study was approved by the ethical review board of General Faculty Hospital Prague. The selection of a representative sample was a crucial step in the research process, as it ensured the validity and reliability of the collected data and allowed for the generalisation of the findings to this population. No participants withdrew from the data collection; no additional invitation and attrition data were available from STEM MARK. This study presents the data from a larger project investigating mental health among young adults in the Czech Republic.

### Instruments

Standardised questionnaires were administered to the participants. Previously validated Czech versions of the Beck Depression Inventory-II (BDI) (44) and the Shirom Melamed Burnout Measure (SMBM) (45) were employed to assess depression and three domains of burnout (emotional, cognitive, and physical), respectively. Furthermore, the Beck Anxiety Inventory (BAI) (46) was used to assess anxiety. The Cognitive Emotion Regulation Questionnaire (CERQ) (47) was used to assess coping strategies. The Adverse Childhood Experiences Questionnaire (ACE) (48, 49) was used to assess traumatic experiences from childhood. The Perceived Stress Scale 10 (PSS-10) (50, 51) was used to assess levels of stress during the past week. The CRAFFT (52, 53) was used to assess risky alcohol use (54). The ENRICHD Social Support Instrument (ESSI) (55) was used to assess social support. In addition, the Buss Perry Aggression Questionnaire Short Form (BPAQ) (56) was used to assess physical and verbal aggression, and the aggression subscale of the Life History of Aggression (LHA) (57) was administered. These measures of aggression were used to create the latent variable for aggressive behaviour. The BDI-II and SMBM were previously validated for the Czech population (44, 45). The remaining instruments (BAI, CERQ, ACE, PSS-10, CRAFFT, ESSI, BPAQ, LHA) were translated into Czech by the research team using forward translation methodology. The translation process prioritized semantic and cultural equivalence while maintaining the psychometric properties. The questionnaires exhibited good internal consistency in our sample, with Cronbach alpha coefficients >.7. The CRAFFT was the only exception, as its Cronbach alpha coefficient was.63 (**Supplementary Table S1** in the **Supplementary Materials**).

### Statistical analysis

All analyses were performed using R software (58). First, chi-square tests and ANOVAs were used to compare sociodemographic characteristics and the variables included in the models between sexes. Structural equation models were estimated using a diagonally weighted least squares estimator in the Lavaan package (59). This estimator was selected because several indicators were Likert-type ordinal item responses and the data did not meet multivariate normality assumptions. This made the use of DWLS framework more appropriate than maximum likelihood estimation. Previous simulation studies suggest that DWLS often performs better than conventional maximum likelihood for ordinal observed variables (60, 61). Model fit was evaluated using multiple indices with commonly used cutoff criteria (62, 63): Comparative Fit Index (CFI ≥.95 indicating good fit), Tucker-Lewis Index (TLI ≥.95), Root Mean Square Error of Approximation (RMSEA ≤.06), and Standardized Root Mean Square Residual (SRMR ≤.08). Values close to these thresholds were considered acceptable fit. The use of multiple fit indices provides a comprehensive evaluation of model adequacy, as different indices are sensitive to different aspects of model misspecification. Mediation analysis was performed by calculating indirect effects via the product of the paths from the predictor to the mediator and from the mediator to the dependent variable. Total effects were calculated by summing the indirect and direct effects.

## Results

**Table 1** shows that there were significant differences between men and women in terms of the highest level of education achieved; the use of cognitive regulation strategies; levels of burnout, stress, depression and anxiety; and the number of ACEs. Descriptive statistics of the sample are shown in **Table 2**.

**Table 1:** Internal consistency of the instruments used.

Scale	Format	Cronbach’s α	Items	N
ACE	Binary yes or no	0.704	10	1027
CRQ Self-Blame	Five point frequency scale	0.817	4	1027
CRQ Acceptance	Five point frequency scale	0.77	4	1027
CRQ Rumination	Five point frequency scale	0.801	4	1027
CRQ Positive Refocusing	Five point frequency scale	0.821	4	1027
CRQ Refocusing and Planning	Five point frequency scale	0.759	4	1027
CRQ Positive Reappraisal	Five point frequency scale	0.85	4	1027
CRQ Putting into Perspective	Five point frequency scale	0.816	4	1027
CRQ Catastrophising	Five point frequency scale	0.791	4	1027
CRQ Blaming Others	Five point frequency scale	0.822	4	1027
LHA Aggression	Six point frequency scale	0.862	5	1027
BPAQ Physical Aggression	Five point agreement scale	0.809	4	1027
BPAQ Verbal Aggression	Five point agreement scale	0.726	3	1027
SMBM Physical	Seven point frequency scale	0.894	6	803
SMBM Cognitive	Seven point frequency scale	0.901	5	803
SMBM Emotional	Seven point frequency scale	0.806	3	803
Social Support	Five point frequency scale	0.915	6	1027
Perceived Stress Scale	5-point frequency scale	0.819	10	1027
CRAFFT (Risky Alcohol use)	Binary yes or no	0.627	6	1027
Beck’s Anxiety Inventory (BAI)	4-point severity scale	0.931	21	1027
Beck’s Depression Inventory II (BDI II)	4-point severity scale	0.93	21	1027

**Table 2:** Descriptive statistics.

Variabe name	Mean/N (sd/%) females	Mean/N (sd/%) males	Mean/N (sd/%) total	Chi-square/F (1, 1025)	P value
Age	25.04 (3.91)	24.01 (3.98)	24.53 (3.98)	17.440	.000
Highest Achieved Education					
Primary	93 (53.14)	82 (46.86)	175 (100.00)	23.6	.0003
High School without graduating	92 (59.74)	62 (40.26)	154 (100.00)		
High School graduate	183 (42.26)	250 (57.74)	433 (100.00)		
Bachelor’s	74 (59.20)	51 (40.80)	125 (100.00)		
Master’s	77 (55.40)	62 (44.60)	139 (100.00)		
Postgraduate Studies	1 (100.00)	0 (0.00)	1 (100.00)		
Monthly Income					
=< 10 000	10 (76.92)	3 (23.08)	13 (100.00)	19.9	.07
10 001–15–000 Kc	14 (77.78)	4 (22.22)	18 (100.00)		
15 001–20–000 Kc	13 (52.00)	12 (48.00)	25 (100.00)		
20 001–25–000 Kc	17 (48.57)	18 (51.43)	35 (100.00)		
25 001–30–000 Kc	36 (49.32)	37 (50.68)	73 (100.00)		
30 001–35–000 Kc	41 (59.42)	28 (40.58)	69 (100.00)		
35 001–40–000 Kc	53 (56.99)	40 (43.01)	93 (100.00)		
40 001–50–000 Kc	91 (53.22)	80 (46.78)	171 (100.00)		
50 001–60–000 Kc	70 (50.36)	69 (49.64)	139 (100.00)		
60 001–70–000 Kc	35 (42.17)	48 (57.83)	83 (100.00)		
70–001 Kc or more	60 (45.80)	71 (54.20)	131 (100.00)		
Don’t want to disclose	40 (41.67)	56 (58.33)	96 (100.00)		
I do not know	40 (49.38)	41 (50.62)	81 (100.00)		
Cognitive Emotional Regulation Strategies					
Self-Blaming	10.38 (3.62)	10.48 (3.51)	10.43 (3.56)	0.200	.658
Acceptance	11.4 (3.43)	11.32 (3.51)	11.36 (3.47)	0.130	.717
Rumination	11.09 (3.68)	10.36 (3.62)	10.73 (3.66)	10.490	.001
Positive Refocusing	10.21 (3.61)	9.94 (3.59)	10.07 (3.6)	1.480	.225
Refocusing and Planning	10.65 (3.41)	11.19 (3.45)	10.92 (3.44)	6.250	.013
Positive Reappraisal	11.02 (3.88)	11.18 (3.85)	11.1 (3.86)	0.460	.496
Putting Things into Perspective	11.5 (3.68)	10.83 (3.71)	11.17 (3.71)	8.300	.004
Catastrophising	8.68 (3.37)	8.36 (3.4)	8.52 (3.39)	2.320	.128
Blaming Others	7.78 (3.13)	8.31 (3.02)	8.04 (3.09)	7.630	.006
Buss-Perry Aggression Questionnaire Short Version					
Physical Aggression	6.36 (3.09)	6.72 (3.17)	6.54 (3.14)	3.510	.061
Verbal Aggression	6.22 (2.75)	6.22 (2.68)	6.22 (2.72)	0.000	.998
Life History of Aggression					
Aggressive Behaviour	7.17 (6.43)	8.13 (6.54)	7.64 (6.5)	5.600	.018
Shirom-Melamed Burnout Measure (n = 487 for Males and n = 316 for Females)					
Physical Burnout	23.87 (7.96)	21.46 (7.67)	22.41 (7.87)	18.410	.000
Cognitive Burnout	17.99 (6.34)	16.8 (6.29)	17.27 (6.33)	6.900	.009
Emotional Burnout	9.38 (4.03)	9.04 (3.86)	9.18 (3.93)	1.430	.231
Social Support	17.85 (5.51)	16.21 (6)	17.04 (5.81)	20.760	.000
Self-Reported Stress	19.76 (6.32)	17.92 (6.25)	18.85 (6.35)	21.850	.000
Risky Alcohol Use	1.41 (1.39)	1.67 (1.49)	1.54 (1.45)	8.160	.004
Beck Anxiety Inventory	13.36 (11.41)	10.64 (10.12)	12.02 (10.88)	16.230	.000
Beck Depression Inventory	15.06 (11.19)	11.81 (10.37)	13.46 (10.91)	23.260	.000
Adverse Childhood Experiences	2.02 (1.89)	1.49 (1.8)	1.76 (1.87)	21.460	.000

The proposed multigroup model had an acceptable fit to the data (CFI = 0.956, TLI = 0.947, RMSEA = 0.06, SRMR = 0.07) based on the aforenoted cut off values (62, 63). The fit indices suggested the presence of configural invariance. However, weak invariance was not present, as the model with constrained factor loadings had a significantly worse fit, chi-square difference (11) = 29.1, p = .002. Given that strong and strict invariance presupposes weak invariance, further model comparisons were not performed. Instead, to avoid biased results, two separate models were run – one for men and one for women. The models showed good fit with the data from males and acceptable fit with the data from females (CFI = 0.961, TLI = 0.953, RMSEA = 0.056, SRMR = 0.068 for males; CFI = 0.949, TLI = 0.938, RMSEA = 0.065, SRMR = 0.081 for females) (62, 63). *Post hoc* power analyses were employed to verify acceptable test power, which was based on the respective RMSEA indices (with an alpha of.05 and a threshold of.9).

**Figure 1:**
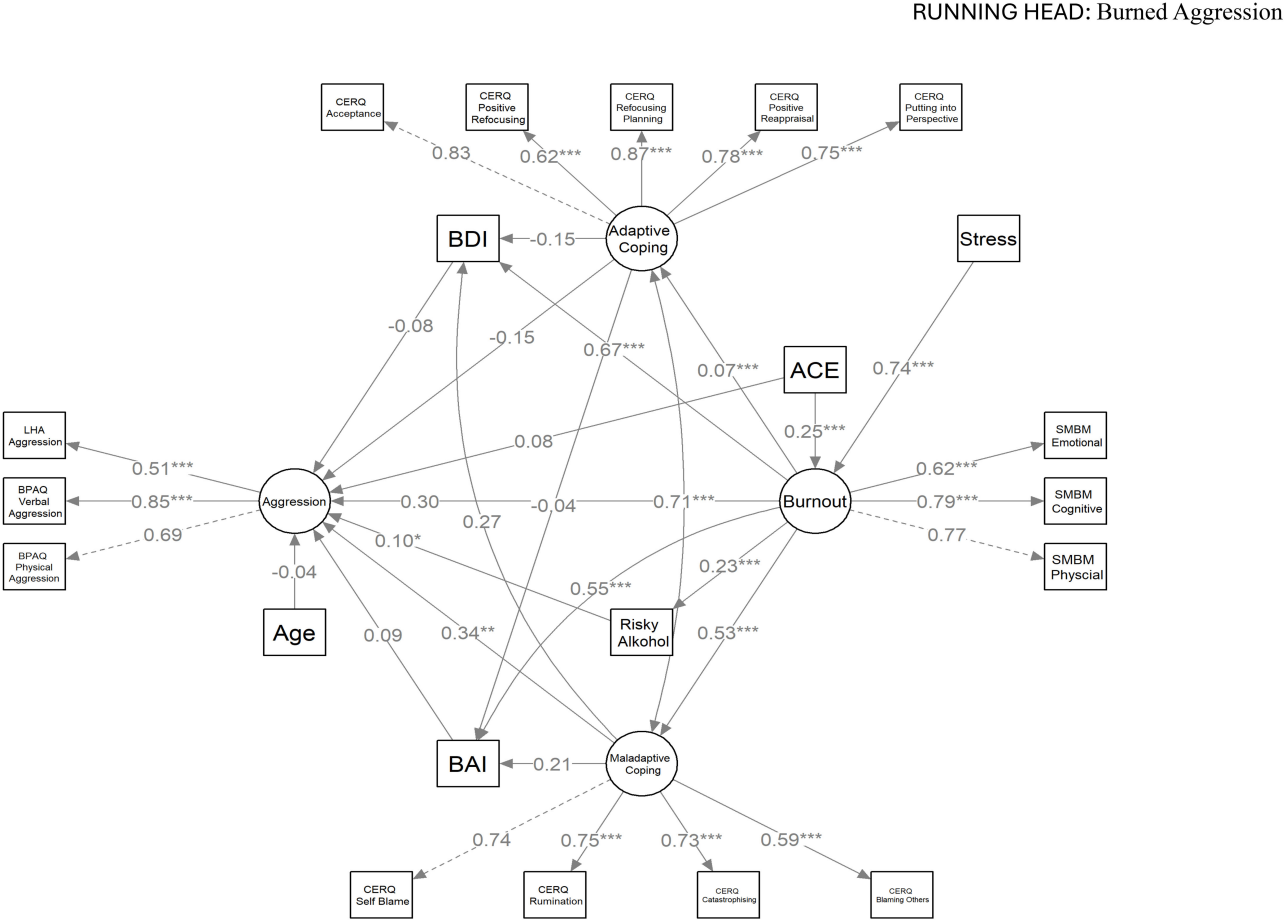
Full model for males.

**Figure 2:**
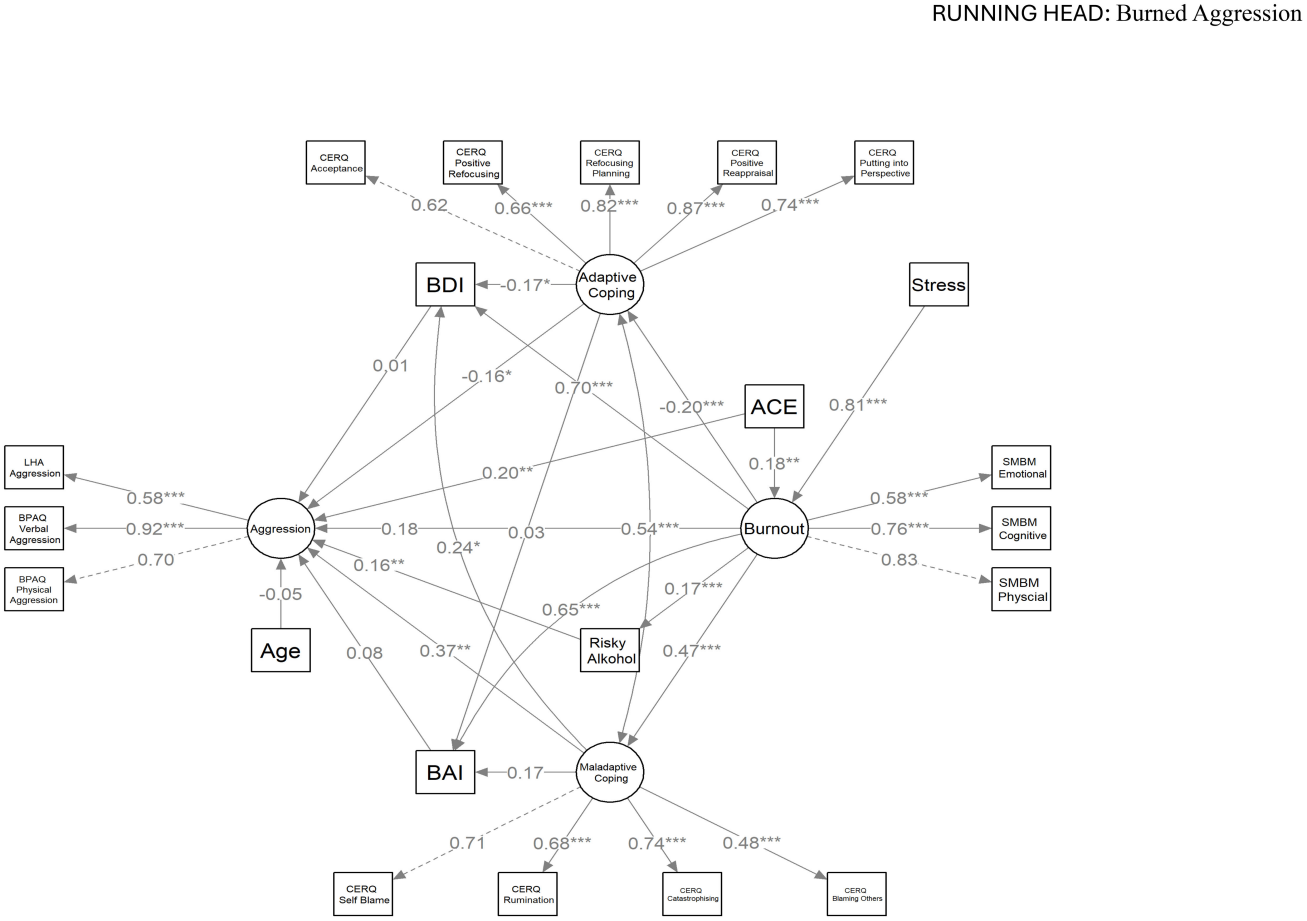
Full model for females.

**Figures 1**, **2** show the standardised factor loadings. The models also included the expected mediating effects of adaptive and maladaptive cognitive regulation strategies and risky alcohol consumption of the effect of burnout on aggressive behaviour. For males, the indirect effect of burnout and adaptive coping strategies was not significant (β= -0.011, p = 0.12); therefore, a model without the mediating factors was run. The factor loadings are presented in **Supplementary Tables S2**, **S3** in the appendix.

Although the relationship between burnout and aggressive behaviour was nonsignificant in males, β= 0.296, p = 0.05, and in female models, β= 0.176, p = 0.469, there were some differences between the sexes. For men, both maladaptive strategies and risky alcohol consumption positively mediated the influence of burnout on aggression (maladaptive strategies: β= 0.184, p = 0.005, and the total effect β= 0.48, p = 0.003; risky alcohol consumption: β= 0.022, p = 0.02, and the total effect β = 0.318, p = 0.034). However, for women, the indirect effects of adaptive strategies (β= 0.031, p = 0.047), maladaptive coping strategies (β = 0.176, p = 0.002), and risky alcohol consumption (β= 0.027, p = 0.001) were significant. Neither the total effect for both regulation strategies (β= 0.383, p = 0.158) nor the total effect for risky alcohol consumption (β = 0.203, p = 0.401) was significant. These findings suggest that maladaptive strategies and risky alcohol consumption only partially mediated the effect of burnout on aggression. Furthermore, an increased number of ACEs was significantly associated with increased aggressive behaviour among women (β = 0.077, p = 0.001) but not among men (β = 0.202, p = 0.001). The effect of burnout also differed between sexes. Males reporting higher levels of burnout showed increased reliance on adaptive emotion regulation strategies (β = 0.071, p <.001); among females, this direction of this relationship was reversed (β = -0.197, p = .001). However, while the use of adaptive regulation strategies was associated with decreased depressive symptoms (β = -0.172, p = 0.039) and lower aggression among women (β = 0.159, p = 0.046), these associations were not significant among men (β = -0.152, p = 0.124 for depressive symptomology and β = -0.153, p = 0.076 for lower aggression). Moreover, reliance on maladaptive strategies was associated with an increase in depressive symptoms among women (β = 0.244, p = 0.032) but not among men (β = 0.271, p = 0.054).

## Discussion

The results showed that although burnout does not have a direct effect on aggressive behaviour among young adults in general, it does have an indirect effect. Among men, burnout was associated with greater reliance on maladaptive emotion regulation strategies and risky alcohol consumption, both of which were associated with physical aggression. Among women, burnout was associated with decreased reliance on adaptive strategies, which led to lower levels of physical aggression. As expected, among both sexes, stress was directly associated with burnout, which was associated with both depression and anxiety. Finally, ACEs were related to higher rates of burnout in both sexes; however, ACEs were inversely associated with aggression among women, while the association was positive among men. Thus, yielded results matched the expectations in part.

The central hypothesis was partially supported, as burnout was indirectly associated with physical aggression. This study extends the findings of Tzeletopoulou et al. (27) to both working and studying young adults. However, depression was not associated with physical aggression. Furthermore, the effect of burnout on aggression was fully mediated by maladaptive strategies and risky alcohol consumption. This finding provides context for the link between burnout and aggression in police officers, as reported by Queirós et al. (28). However, the fully mediated model was only observed among males.

Meanwhile, the direct positive effect of maladaptive strategies were on physical aggression followed the existing literature (34, 35). This allowed to extend the mediating effect of maladaptive strategies on the relationship between stress and aggression (22) to burnout. However, the association between adaptive strategies and physical aggression was only observed among women. Burnout can facilitate reliance on maladaptive coping, thereby amplifying aggression among both sexes. However, increased reliance on adaptive coping due to this exhaustive state can reduce levels of aggression among women. Thus, while maladaptive emotion regulation induced by burnout can serve as an impellent (64) or route (3) for aggression, adaptive regulation functions as an inhibitor among women. In practice, this suggests that there should be a higher priority on reducing reliance on maladaptive coping in anti-aggression and burnout interventions. However, there were additional factors relevant to physical aggression.

The observed sex differences in emotion regulation pathways warrant further consideration. Women in our sample showed greater reliance on adaptive emotion regulation strategies under normal conditions, but burnout reversed this pattern, leading to decreased use of these strategies. This differential effect may explain why adaptive coping protected against aggression in women but not in men. Conversely, maladaptive coping strategies (34) and alcohol consumption (36) emerged as primary pathways to aggression for both sexes under burnout conditions, though the total effects differed between groups. These findings suggest that burnout prevention programs should account for these sex-specific regulatory pathways when designing interventions.

The mediating role of risky alcohol consumption was confirmed among both sexes. This showed that burnout can contribute to increased alcohol use (38). The results also revealed that burnout is associated with increased alcohol consumption among young adults in both the workplace and the academic context (39, 40). Furthermore, the models support the established link between alcohol and aggression (36), indicating that burnout combined with increased alcohol consumption can drive aggression (3, 31). This, in turn, raises the importance of determining whether alcohol abuse is present as a typical coping mechanism when designing burnout interventions.

Two factors related to burnout but not aggression were depression and anxiety for both sexes. This result is consistent with previous findings among working adults (16) and extends the findings to young adults who either work or study. However, in women, reliance on both adaptive and maladaptive strategies was associated with higher levels of depressive symptoms. The unexpected nature of this finding highlights the relativity of the “adaptiveness” of emotion regulation strategies. Most likely, the adopted composition of adaptive coping factors does not offer protection against depression. As a results, this finding once again highlights the need of an individualised approach when dealing with clients.

Lastly, in addition to the suggested role of ACEs in burnout among health professionals and nursing students (42, 43), the current study revealed that ACEs are also associated with burnout among young adults in different occupations. Moreover, the models suggested that ACEs are associated with aggression not only among the forensic male population (65), but also in the community population of young adult men. Interestingly, this association was inverse, raising the possibility of different mechanisms of aggressive behaviour between sexes.

However, the current study has several limitations. SEM cannot be used to identify the specific maladaptive coping strategies that mediate the effect of burnout on aggression. However, as this was the first analysis involving a representative sample of Czech young adults, an initial investigation of latent variables was performed. Future studies should examine the specific coping strategies that can mediate the effect of burnout on aggression. Although the included sample was representative, due to the integral connections between burnout and working or studying, some respondents had to be excluded from the analysis. As expected, most of the excluded individuals were women, thus leading to unequal sample sizes for the two models. This also led to the model being relatively simply and only including a few of the relevant variables.

Furthermore, the cross-sectional design precludes causal inferences and the establishment of temporal relationships between burnout and aggressive behaviour. While the theoretical framework and existing literature support the hypothesized directional relationships, longitudinal research is necessary to confirm the temporal sequence and rule out reverse causality. Likewise, the reliance on self-report measures introduces potential for social desirability bias and retrospective recall limitations. Lastly, while we controlled for several demographic variables and included multiple theoretically relevant constructs, potential residual confounding from unmeasured variables cannot be ruled out. The models could not include all potentially relevant variables such as social support quality or specific occupational stressors, which may moderate the relationships examined here.

## Conclusion

The results of the present study are twofold. First, among Czech young adults, increasing levels of burnout are associated with increased reliance on maladaptive emotion regulation strategies and alcohol consumption, thereby facilitating physical aggression. Thus, maladaptive emotion regulation strategies and alcohol consumption are considered to be additional negative consequences of burnout among young adults. However, the findings also highlighted the importance of promoting adaptive coping strategies to prevent aggressive behaviour. Second, the comparison of SEMs between men and women demonstrated that the specific mechanisms facilitating aggressive behaviour differ between sexes. Adaptive emotion regulation strategies can prevent aggression among women but not among men. However, adaptive emotion regulation strategies increase depressive symptoms only among women.

## Data Availability

The raw data supporting the conclusions of this article will be made available by the authors, without undue reservation.
