# Supplementary material for: Burned aggression: the relationship between burnout and aggressive behaviour among young adults in Czechia
Source: Front Psychiatry. 2026 Jul 1;17:1872129. doi: 10.3389/fpsyt.2026.1872129 (PMC13368932; doi:10.3389/fpsyt.2026.1872129)
Supplement: Supplementary file 1 [file Table1.docx]

| **Table A1. Internal Consistency of Questionnaires** | |  |
| --- | --- | --- |
| **name** | **Cronbach’s alpha** | |
| ACE | 0.70 | |
| CRQ Self Blame | 0.82 | |
| CRQ Acceptance | 0.77 | |
| CRQ Rumination | 0.80 | |
| CRQ Positive Refocusing | 0.82 | |
| CRQ Refocusing and Planning | 0.76 | |
| CRQ Positive Reappraising | 0.85 | |
| CRQ Putting things into Perspective | 0.82 | |
| CRQ Catastrophising | 0.79 | |
| CRQ Blaming Others | 0.82 | |
| LHA Aggression | 0.86 | |
| BPAQ Physical Aggression | 0.81 | |
| BPAQ Verbal Aggression | 0.73 | |
| SMBM Physical | 0.89 | |
| SMBM Cognitive | 0.90 | |
| SMBM Emotional | 0.81 | |
| Social Support | 0.91 | |
| Stress | 0.82 | |
| CRAFFT | 0.63 | |
| BAI | 0.93 | |
| BDI | 0.93 | |
