# Supplementary material for: Burned aggression: the relationship between burnout and aggressive behaviour among young adults in Czechia
Source: Front Psychiatry. 2026 Jul 1;17:1872129. doi: 10.3389/fpsyt.2026.1872129 (PMC13368932; doi:10.3389/fpsyt.2026.1872129)
Supplement: Supplementary file 2 [file Table2.docx]

| Table A2. Full loadings for the model fitted to males | | | | | | |  |
| --- | --- | --- | --- | --- | --- | --- | --- |
| **Outcome** | **Predictor** | **Relationship** | **Standardised Estimate** | **S.E.** | **p value** | **Lower CI** | **Upper CI** |
| Adaptive Coping | =~ | CERQ Acceptance | 0.825 | 0.041 | 0.000 | 0.745 | 0.906 |
| Adaptive Coping | =~ | CERQ Positive Refocusing | 0.616 | 0.035 | 0.000 | 0.546 | 0.685 |
| Adaptive Coping | =~ | CERQ Refocusing Planning | 0.868 | 0.044 | 0.000 | 0.782 | 0.954 |
| Adaptive Coping | =~ | CERQ Positive Reappraisal | 0.776 | 0.039 | 0.000 | 0.700 | 0.852 |
| Adaptive Coping | =~ | CERQ Putting into Perspective | 0.752 | 0.039 | 0.000 | 0.676 | 0.828 |
| Maladaptive Coping | =~ | CERQ Self Blame | 0.739 | 0.037 | 0.000 | 0.666 | 0.813 |
| Maladaptive Coping | =~ | CERQ Rumination | 0.748 | 0.038 | 0.000 | 0.674 | 0.823 |
| Maladaptive Coping | =~ | CERQ Catastrophising | 0.732 | 0.039 | 0.000 | 0.655 | 0.809 |
| Maladaptive Coping | =~ | CERQ Blaming Others | 0.590 | 0.035 | 0.000 | 0.520 | 0.659 |
| Aggression | =~ | BPAQ Physical Aggression | 0.695 | 0.049 | 0.000 | 0.599 | 0.790 |
| Aggression | =~ | BPAQ Verbal Aggression | 0.849 | 0.050 | 0.000 | 0.752 | 0.947 |
| Aggression | =~ | LHA Aggression | 0.510 | 0.034 | 0.000 | 0.443 | 0.577 |
| Burnout | =~ | SMBM Physical | 0.771 | 0.037 | 0.000 | 0.699 | 0.843 |
| Burnout | =~ | SMBM Cognitive | 0.785 | 0.037 | 0.000 | 0.713 | 0.858 |
| Burnout | =~ | SMBM Emotional | 0.619 | 0.032 | 0.000 | 0.555 | 0.682 |
| BDI | ~ | Burnout | 0.666 | 0.094 | 0.000 | 0.482 | 0.850 |
| BDI | ~ | Maladaptive Coping | 0.271 | 0.140 | 0.054 | -0.004 | 0.546 |
| BDI | ~ | Adaptive Coping | -0.152 | 0.099 | 0.124 | -0.345 | 0.042 |
| BAI | ~ | Burnout | 0.547 | 0.085 | 0.000 | 0.381 | 0.714 |
| BAI | ~ | Maladaptive Coping | 0.208 | 0.129 | 0.105 | -0.043 | 0.460 |
| BAI | ~ | Adaptive Coping | -0.044 | 0.091 | 0.627 | -0.223 | 0.134 |
| Burnout | ~ | ACE | 0.249 | 0.047 | 0.000 | 0.156 | 0.342 |
| Burnout | ~ | Stress | 0.744 | 0.041 | 0.000 | 0.664 | 0.824 |
| Aggression | ~ | Burnout | 0.296 | 0.151 | 0.050 | 0.000 | 0.591 |
| Aggression | ~ | Maladaptive Coping | 0.345 | 0.122 | 0.005 | 0.105 | 0.584 |
| Aggression | ~ | Adaptive Coping | -0.153 | 0.086 | 0.076 | -0.322 | 0.016 |
| Aggression | ~ | Risky Alcohol Use | 0.097 | 0.043 | 0.025 | 0.012 | 0.182 |
| Aggression | ~ | ACE | 0.077 | 0.051 | 0.135 | -0.024 | 0.178 |
| Aggression | ~ | BDI | -0.083 | 0.151 | 0.582 | -0.380 | 0.213 |
| Aggression | ~ | BAI | 0.094 | 0.079 | 0.234 | -0.061 | 0.249 |
| Aggression | ~ | Age | -0.036 | 0.041 | 0.379 | -0.115 | 0.044 |
| Maladaptive Coping | ~ | Burnout | 0.535 | 0.027 | 0.000 | 0.481 | 0.588 |
| Adaptive Coping | ~ | Burnout | 0.071 | 0.017 | 0.000 | 0.038 | 0.105 |
| Risky Alcohol Use | ~ | Burnout | 0.228 | 0.025 | 0.000 | 0.179 | 0.277 |
| Adaptive Coping | ~~ | Maladaptive Coping | 0.713 | 0.040 | 0.000 | 0.635 | 0.791 |
| CERQ Acceptance | ~~ | CERQ Acceptance | 0.319 | 0.068 | 0.000 | 0.185 | 0.452 |
| CERQ Positive Refocusing | ~~ | CERQ Positive Refocusing | 0.621 | 0.044 | 0.000 | 0.536 | 0.707 |
| CERQ Refocusing Planning | ~~ | CERQ Refocusing Planning | 0.247 | 0.076 | 0.001 | 0.098 | 0.395 |
| CERQ Positive Reappraisal | ~~ | CERQ Positive Reappraisal | 0.398 | 0.060 | 0.000 | 0.280 | 0.516 |
| CERQ Putting into Perspective | ~~ | CERQ Putting into Perspective | 0.434 | 0.058 | 0.000 | 0.321 | 0.548 |
| CERQ Self Blame | ~~ | CERQ Self Blame | 0.453 | 0.055 | 0.000 | 0.345 | 0.562 |
| CERQ Rumination | ~~ | CERQ Rumination | 0.440 | 0.057 | 0.000 | 0.328 | 0.552 |
| CERQ Catastrophising | ~~ | CERQ Catastrophising | 0.464 | 0.058 | 0.000 | 0.351 | 0.577 |
| CERQ Blaming Others | ~~ | CERQ Blaming Others | 0.652 | 0.042 | 0.000 | 0.570 | 0.734 |
| BPAQ Physical Aggression | ~~ | BPAQ Physical Aggression | 0.517 | 0.068 | 0.000 | 0.385 | 0.650 |
| BPAQ Verbal Aggression | ~~ | BPAQ Verbal Aggression | 0.279 | 0.085 | 0.001 | 0.113 | 0.445 |
| LHA Aggression | ~~ | LHA Aggression | 0.740 | 0.035 | 0.000 | 0.672 | 0.808 |
| SMBM Physical | ~~ | SMBM Physical | 0.406 | 0.057 | 0.000 | 0.295 | 0.517 |
| SMBM Cognitive | ~~ | SMBM Cognitive | 0.383 | 0.058 | 0.000 | 0.269 | 0.497 |
| SMBM Emotional | ~~ | SMBM Emotional | 0.617 | 0.040 | 0.000 | 0.539 | 0.696 |
| BDI | ~~ | BDI | 0.334 | 0.078 | 0.000 | 0.181 | 0.487 |
| BAI | ~~ | BAI | 0.548 | 0.054 | 0.000 | 0.442 | 0.655 |
| Risky Alcohol Use | ~~ | Risky Alcohol Use | 0.948 | 0.011 | 0.000 | 0.926 | 0.971 |
| Adaptive Coping | ~~ | Adaptive Coping | 0.995 | 0.002 | 0.000 | 0.990 | 1.000 |
| Maladaptive Coping | ~~ | Maladaptive Coping | 0.714 | 0.029 | 0.000 | 0.657 | 0.771 |
| Aggression | ~~ | Aggression | 0.659 | 0.039 | 0.000 | 0.582 | 0.735 |
| Burnout | ~~ | Burnout | 0.308 | 0.061 | 0.000 | 0.188 | 0.428 |
| ACE | ~~ | ACE | 1.000 | 0.000 | NA | 1.000 | 1.000 |
| ACE | ~~ | Stress | 0.206 | 0.048 | 0.000 | 0.111 | 0.300 |
| ACE | ~~ | Age | -0.079 | 0.045 | 0.081 | -0.167 | 0.010 |
| Stress | ~~ | Stress | 1.000 | 0.000 | NA | 1.000 | 1.000 |
| Stress | ~~ | Age | -0.221 | 0.030 | 0.000 | -0.279 | -0.163 |
| Age | ~~ | Age | 1.000 | 0.000 | NA | 1.000 | 1.000 |
| Maladaptive Coping Indirect Effect | := | a1*b1 | 0.184 | 0.066 | 0.005 | 0.055 | 0.314 |
| Risky Drinking Indirect Effect | := | a3*b3 | 0.022 | 0.010 | 0.020 | 0.003 | 0.041 |
| Total Effect Coping | := | c1+(a1*b1) | 0.480 | 0.164 | 0.003 | 0.159 | 0.800 |
| Total Effect Risky Drinking | := | c1+(a3*b3) | 0.318 | 0.150 | 0.034 | 0.024 | 0.611 |
| =~ latent variable loading, ~ regression coefficient, ~~ (co)variance, := specified paths | | | | | | | |
