# Supplementary material for: Burned aggression: the relationship between burnout and aggressive behaviour among young adults in Czechia
Source: Front Psychiatry. 2026 Jul 1;17:1872129. doi: 10.3389/fpsyt.2026.1872129 (PMC13368932; doi:10.3389/fpsyt.2026.1872129)
Supplement: Supplementary file 3 [file Table3.docx]

| Table A3. Full loadings for the model fitted to females | | | | | | |  |
| --- | --- | --- | --- | --- | --- | --- | --- |
| **Outcome** | **Relationship** | **Predictor** | **Standardised Estimate** | **S.E.** | **p value** | **Lower CI** | **Upper CI** |
| Adaptive Coping | =~ | CERQ Acceptance | 0.619 | 0.044 | 0.000 | 0.532 | 0.706 |
| Adaptive Coping | =~ | CERQ Positive Refocusing | 0.657 | 0.047 | 0.000 | 0.565 | 0.749 |
| Adaptive Coping | =~ | CERQ Refocusing Planning | 0.825 | 0.053 | 0.000 | 0.721 | 0.929 |
| Adaptive Coping | =~ | CERQ Positive Reappraisal | 0.872 | 0.053 | 0.000 | 0.769 | 0.975 |
| Adaptive Coping | =~ | CERQ Putting into Perspective | 0.742 | 0.047 | 0.000 | 0.650 | 0.834 |
| Maladaptive Coping | =~ | CERQ Self Blame | 0.706 | 0.046 | 0.000 | 0.617 | 0.796 |
| Maladaptive Coping | =~ | CERQ Rumination | 0.682 | 0.044 | 0.000 | 0.597 | 0.767 |
| Maladaptive Coping | =~ | CERQ Catastrophising | 0.737 | 0.051 | 0.000 | 0.636 | 0.837 |
| Maladaptive Coping | =~ | CERQ Blaming Others | 0.482 | 0.043 | 0.000 | 0.398 | 0.567 |
| Aggression | =~ | BPAQ Physical Aggression | 0.702 | 0.060 | 0.000 | 0.584 | 0.819 |
| Aggression | =~ | BPAQ Verbal Aggression | 0.918 | 0.061 | 0.000 | 0.799 | 1.038 |
| Aggression | =~ | LHA Aggression | 0.585 | 0.041 | 0.000 | 0.504 | 0.665 |
| Burnout | =~ | SMBM Physical | 0.828 | 0.044 | 0.000 | 0.742 | 0.914 |
| Burnout | =~ | SMBM Cognitive | 0.756 | 0.042 | 0.000 | 0.673 | 0.839 |
| Burnout | =~ | SMBM Emotional | 0.579 | 0.039 | 0.000 | 0.503 | 0.655 |
| BDI | ~ | Burnout | 0.699 | 0.097 | 0.000 | 0.509 | 0.889 |
| BDI | ~ | Maladaptive Coping | 0.244 | 0.114 | 0.032 | 0.021 | 0.468 |
| BDI | ~ | Adaptive Coping | -0.172 | 0.083 | 0.039 | -0.334 | -0.009 |
| BAI | ~ | Burnout | 0.649 | 0.093 | 0.000 | 0.467 | 0.832 |
| BAI | ~ | Maladaptive Coping | 0.167 | 0.107 | 0.118 | -0.042 | 0.376 |
| BAI | ~ | Adaptive Coping | 0.032 | 0.079 | 0.690 | -0.124 | 0.187 |
| Burnout | ~ | ACE | 0.176 | 0.060 | 0.003 | 0.059 | 0.293 |
| Burnout | ~ | Stress | 0.813 | 0.047 | 0.000 | 0.720 | 0.905 |
| Aggression | ~ | Burnout | 0.176 | 0.243 | 0.469 | -0.300 | 0.651 |
| Aggression | ~ | Maladaptive Coping | 0.371 | 0.114 | 0.001 | 0.148 | 0.595 |
| Aggression | ~ | Adaptive Coping | -0.159 | 0.080 | 0.046 | -0.315 | -0.003 |
| Aggression | ~ | Risky Alcohol Use | 0.164 | 0.051 | 0.001 | 0.065 | 0.263 |
| Aggression | ~ | ACE | 0.202 | 0.062 | 0.001 | 0.080 | 0.324 |
| Aggression | ~ | BDI | 0.007 | 0.253 | 0.978 | -0.489 | 0.503 |
| Aggression | ~ | BAI | 0.077 | 0.129 | 0.548 | -0.175 | 0.330 |
| Aggression | ~ | Age | -0.046 | 0.056 | 0.416 | -0.155 | 0.064 |
| Maladaptive Coping | ~ | Burnout | 0.474 | 0.033 | 0.000 | 0.409 | 0.539 |
| Adaptive Coping | ~ | Burnout | -0.197 | 0.023 | 0.000 | -0.243 | -0.151 |
| Risky Alcohol Use | ~ | Burnout | 0.166 | 0.028 | 0.000 | 0.112 | 0.221 |
| Adaptive Coping | ~~ | Maladaptive Coping | 0.539 | 0.047 | 0.000 | 0.447 | 0.631 |
| CERQ Acceptance | ~~ | CERQ Acceptance | 0.616 | 0.055 | 0.000 | 0.509 | 0.724 |
| CERQ Positive Refocusing | ~~ | CERQ Positive Refocusing | 0.569 | 0.062 | 0.000 | 0.448 | 0.690 |
| CERQ Refocusing Planning | ~~ | CERQ Refocusing Planning | 0.320 | 0.087 | 0.000 | 0.149 | 0.491 |
| CERQ Positive Reappraisal | ~~ | CERQ Positive Reappraisal | 0.240 | 0.092 | 0.009 | 0.060 | 0.419 |
| CERQ Putting into Perspective | ~~ | CERQ Putting into Perspective | 0.450 | 0.070 | 0.000 | 0.313 | 0.586 |
| CERQ Self Blame | ~~ | CERQ Self Blame | 0.501 | 0.065 | 0.000 | 0.375 | 0.628 |
| CERQ Rumination | ~~ | CERQ Rumination | 0.535 | 0.059 | 0.000 | 0.419 | 0.651 |
| CERQ Catastrophising | ~~ | CERQ Catastrophising | 0.458 | 0.075 | 0.000 | 0.310 | 0.605 |
| CERQ Blaming Others | ~~ | CERQ Blaming Others | 0.767 | 0.042 | 0.000 | 0.686 | 0.849 |
| BPAQ Physical Aggression | ~~ | BPAQ Physical Aggression | 0.508 | 0.084 | 0.000 | 0.343 | 0.673 |
| BPAQ Verbal Aggression | ~~ | BPAQ Verbal Aggression | 0.157 | 0.112 | 0.162 | -0.063 | 0.376 |
| LHA Aggression | ~~ | LHA Aggression | 0.658 | 0.048 | 0.000 | 0.564 | 0.752 |
| SMBM Physical | ~~ | SMBM Physical | 0.315 | 0.073 | 0.000 | 0.172 | 0.458 |
| SMBM Cognitive | ~~ | SMBM Cognitive | 0.429 | 0.064 | 0.000 | 0.303 | 0.554 |
| SMBM Emotional | ~~ | SMBM Emotional | 0.664 | 0.045 | 0.000 | 0.577 | 0.752 |
| BDI | ~~ | BDI | 0.245 | 0.088 | 0.005 | 0.073 | 0.416 |
| BAI | ~~ | BAI | 0.451 | 0.071 | 0.000 | 0.312 | 0.589 |
| Risky Alcohol Use | ~~ | Risky Alcohol Use | 0.972 | 0.009 | 0.000 | 0.954 | 0.990 |
| Adaptive Coping | ~~ | Adaptive Coping | 0.961 | 0.009 | 0.000 | 0.943 | 0.979 |
| Maladaptive Coping | ~~ | Maladaptive Coping | 0.776 | 0.031 | 0.000 | 0.714 | 0.837 |
| Aggression | ~~ | Aggression | 0.539 | 0.056 | 0.000 | 0.429 | 0.650 |
| Burnout | ~~ | Burnout | 0.248 | 0.075 | 0.001 | 0.101 | 0.395 |
| ACE | ~~ | ACE | 1.000 | 0.000 | NA | 1.000 | 1.000 |
| ACE | ~~ | Stress | 0.210 | 0.060 | 0.000 | 0.093 | 0.327 |
| ACE | ~~ | Age | -0.140 | 0.056 | 0.013 | -0.250 | -0.029 |
| Stress | ~~ | Stress | 1.000 | 0.000 | NA | 1.000 | 1.000 |
| Stress | ~~ | Age | -0.374 | 0.035 | 0.000 | -0.443 | -0.304 |
| Age | ~~ | Age | 1.000 | 0.000 | NA | 1.000 | 1.000 |
| Maladaptive Coping Indirect Effect | := | a1*b1 | 0.176 | 0.056 | 0.002 | 0.067 | 0.285 |
| Adaptive Coping Indirect Effect | := | a2*b2 | 0.031 | 0.016 | 0.047 | 0.000 | 0.062 |
| Risky Drinking Indirect Effect | := | a3*b3 | 0.027 | 0.009 | 0.001 | 0.011 | 0.044 |
| Total Effect Coping | := | c1+(a1b1)+(a2b2) | 0.383 | 0.272 | 0.158 | -0.149 | 0.915 |
| Total Effect Risky Drinking | := | c1+(a3*b3) | 0.203 | 0.242 | 0.401 | -0.271 | 0.677 |
| =~ latent variable loading, ~ regression coefficient, ~~ (co)variance, := specified paths | | | | | | | |
